# Two assessments to evaluate imagery ability: translation, test-retest reliability and concurrent validity of the German KVIQ and Imaprax

**DOI:** 10.1186/1471-2288-12-127

**Published:** 2012-08-20

**Authors:** Corina Schuster, Anina Lussi, Brigitte Wirth, Thierry Ettlin

**Affiliations:** 1 Reha Rheinfelden, Salinenstrasse 98, Rheinfelden, 4310, Switzerland; 2 Faculty of Health and Life Sciences, Oxford Brookes University, Oxford, United Kingdom; 3 School of Occupational Education (BFS), Winterthur, Switzerland; 4 Institute of Human Movement Sciences and Sport, Zurich, ETH Zurich, Switzerland; 5 Department of Behavioural Neurology, Medical Faculty, University of Basel, Basel, Switzerland

## Abstract

**Background:**

A combination of physical practice and motor imagery (MI) can improve motor function. It is essential to assess MI vividness in patients with sensorimotor impairments before implementing MI interventions. The study's aims were to translate the Canadian Kinaesthetic and Visual Imagery Questionnaire (KVIQ) and the French Imaprax, and to examine reliability and validity of the German versions.

**Methods:**

Questionnaires were translated according to guidelines. With examiner’s help patients (diagnosis: stroke: subacute/chronic, brain tumour, Multiple Sclerosis, Parkinson’s disease) were tested twice within seven days (T0, T1). KVIQ-G: Patients were shown a movement by the examiner, before executing and imagining the movement. They rated vividness of the image and intensity of the sensations on a five-point Likert-scale. Imaprax required a 3-step procedure: imagination of one of six gestures; evaluation of gesture understanding, vividness, and imagery perspective. Questionnaire data were analysed overall and for each group. Reliability parameters were calculated: intraclass correlation coefficient (ICC), Cronbach's alpha, standard error of measurement, minimal detectable change. Validity parameters included Spearman's rank correlation coefficient and factor analysis of the KVIQ-G-20.

**Results:**

Patients (N = 73, 28 females, age: 63 ± 13) showed the following at T0: KVIQ-G-20_vis_ 41.7 ± 9, KVIQ-G-10_vis_ 21.1 ± 5. ICC for KVIQ-G-20_vis_ and KVIQ-G-10_vis_ was 0.77; KVIQ-G-20_kin_ 36.4 ± 12, KVIQ-G-10_kin_ 18.3 ± 6. ICCs for KVIQ-G-20_kin_ and KVIQ-G-10_kin_ were 0.83/0.85; Imaprax_vis_ 32.7 ± 4 and ICC 0.51. Internal consistency was estimated for KVIQ-G-20 α_vis_ = 0.94/α_kin_ = 0.92, KVIQ-G-10 α_vis_ = 0.88/α_kin_ = 0.96, Imaprax-G α_vis_ = 0.70. Validity testing was performed with 19 of 73 patients, who chose an internal perspective: r_s_ = 0.36 (p = 0.13). Factor analysis revealed two factors correlating with r = 0.36. Both explain 69.7% of total variance.

**Conclusions:**

KVIQ-G and Imaprax-G are reliable instruments to assess MI in patients with sensorimotor impairments confirmed by a KVIQ-G-factor analysis. KVIQ-G visual values were higher than kinaesthetic values. Patients with Multiple Sclerosis showed the lowest, subacute stroke patients the highest values. Hemiparetic patients scored lower in both KVIQ-G subscales on affected side compared to non-affected side. It is suggested to administer the Imaprax-G before the KVIQ-G to test patient’s ability to distinguish between external and internal MI perspective. Duration of both questionnaires lead to an educational effect. Imaprax validity testing should be repeated.

## Background

Motor imagery (MI) has been defined as a dynamic state during which the representation of a given motor act is internally rehearsed without motor output by Decety and Grezès in 1999 [1]. It has been shown to be beneficial in motor function recovery for patients after a lesion of the central nervous system (CNS) if MI is added to physical practice (PP). Positive effects of MI have been summarised in several literature reviews [2,3]. To determine participant benefits of MI, it is important to evaluate MI vividness and its changes during a MI intervention period. In a recent literature review MI interventions have been evaluated regarding their MI training session elements and temporal parameters to determine successful MI interventions [4]. Only 41 out of the 141 MI interventions used one or more assessments to evaluate participants’ imagery ability. In particular, in Medicine MI assessments have been used in 11 out of 37 MI interventions. Out of the 11 MI interventions nine were performed with patients after a central lesion, e.g. stroke. Three of the nine MI interventions used the Movement Imagery Questionnaire (MIQ) [5,6] or the Revised Movement Imagery Questionnaire (MIQ-R) [7]. Others used custom-made imagery questionnaires or the Kinesthetic and Visual Imagery Questionnaire (KVIQ) [8]. Both assessments (MIQ and KVIQ) aim to assess MI vividness for motor performance. The MIQ includes 18 items to imagine with different levels of movements, e.g. standing on one leg, jumping straight up in the air, or moving the extended arm. Furthermore, all items have to be imagined using two imagery types: visual MI (9 items) and kinaesthetic MI (9 items). All items are rated on a 7-point scale with 1 representing the lowest quality of seeing or feeling the movement.

Hall and Martin (1997) developed the MIQ for healthy participants [9]. Therefore, movements to be imagined in the MIQ do not consider patient condition, e.g. a stroke patient with a hemiparesis would be asked to imagine her/himself jumping straight up in the air. Malouin and colleagues (2007) developed the KVIQ specifically to address participants with sensorimotor impairments [8]. In particular, KVIQ has been evaluated in patients after stroke, with a lower limb amputation, with an acquired blindness, lower limb immobilisation, and Parkinson’s disease [8,10]. Participants are asked to imagine movements of the dominant and non-dominant body side from the internal perspective. Imagination is once performed using visual MI to see, and once using kinaesthetic MI to feel the movement. Imagined movements include all four limbs and the whole body. All movements are imagined to be performed in a sitting position and in one joint axis. If patients cannot perform the required movement, the examiner will assist to move the patient’s limb or will move it passively.

The two main differences between KVIQ and MIQ are administration and the selection of the movements to be imagined. Whereas the MIQ is self-administered and focuses on complex high-level body movements, the KVIQ requires an examiner to be present, giving the instructions, performing example movements, and filling in the scoring sheet. The KVIQ focuses on simple, one joint axis movements of the upper and lower limbs, head, and trunk in a sitting position.

A further option to test participants’ MI vividness is the computer and video-based Imaprax assessment. Imaprax was developed based on the Imagix software to specifically assess MI vividness in patients after stroke with apraxia by Fournier (2000). It is based on software used with skydivers [11,12]. In a three step procedure patients’ understanding of the movement to be performed, MI vividness, and MI perspective are evaluated in six activities of daily living (ADL) in patients with apraxia following stroke. Imaprax requires an examiner to be present for explanation and operating the software. KVIQ and Imaprax are available in Canadian English and French, respectively.

The aims of this project were 1) to translate both original KVIQ versions (KVIQ-10, KVIQ-20) and Imaprax into the German language, and 2) evaluate their test retest reliability and concurrent validity of the visual imagery subscales in patients with a lesion of the central nervous system, in particular, patients after stroke, with Multiple Sclerosis, and Parkinson’s disease. To allow the administration of both assessments in different patient groups, it is intended to present subgroup and total study population analyses. These analyses enable result comparisons among groups and to the total study population.

## Methods

### MI assessments used

Patients’ MI vividness was assessed with the **Imaprax** software (version 1.1) and the KVIQ. Patients were seated in front of a laptop to watch the Imaprax videos. The software showed the patient instructions on the screen and was operated by the therapist. Patients can read them themselves or they can be read aloud by the therapist. The instructions were formulated in a standardised way for all six gestures, e.g. “Imagine with closed eyes that you applaud.” Six complex unilateral and bilateral multi-joint upper limb gestures or activities of daily living (Table 1) were evaluated in a standardised three step procedure: patients were asked 1) to select the correct gesture or activity from three proposed ones, 2) to evaluate the vividness of their ‘inner picture’, and 3) to determine the internal (imagine the movement as watched through own eyes) or external (imagine the movement as standing in a corner and watching the imagined person from a distance performing the movement) perspective used for their ‘inner picture’. During step 2, patients were presented five videos showing the same person performing the same gesture but in different vividness levels. The video could be selected by clicking the respective button below the video frame. Original videos (altered in contrast and luminosity) from the French Imaprax version were used. Additionally, patients were offered two options to rate their ‘inner picture’ as more or less vivid than in the watched videos. In total, vividness could be rated on a 7-point scale. The total score of the visual subscale can range between 6 to 42 points.

**Table 1:** Patients’ chosen perspective for the Imaprax evaluation tool at T0 and T1

**Actions mentally performed using an internal perspective**	Subacute group N = 17*	**Chronic group N = 34**	**Left parietal lob involved N = 7**	**Multiple Sclerosis N = 7**	**Parkinson’s disease N = 8**	**Total N = 73**
To beckon somebody	4/7	9/7	2/2	2/1	4/4	21/21
To cut something	9/9	14/17	4/5	3/2	6/4	36/37
To write something	7/6	16/17	2/4	2/3	4/5	31/35
To brush one’s teeth	4/5	7/8	1/2	3/2	3/3*	18/20
To cock a snook	5/5	8/9	2*/3	2/2	3/3	20*/22*
To applaud somebody	7/7	15/17	4/3	4/2	3/3	33/32*

**Legend:** N = sample size, * = data of one patient missing.

During step 3, patients were presented two videos showing the same person performing the same gesture from the internal and the external imagery perspective. After watching both videos patients determine, which of the offered perspective represents the perspective they have used to imagine the gesture.

The **KVIQ** includes a visual and a kinaesthetic imagery scale [8]. The questionnaire is available in a short (10 items) and a long version (20 items). All requested movements for the visual and the kinaesthetic imagery scale of the KVIQ have to be imagined from the internal perspective once per scale only. The KVIQ gives standardised instructions for each movement performance and its evaluation in a test manual. Firstly, for the visual imagery ability evaluation patients have to concentrate on the clarity of the image during the imagination of all movements (original instructions from the Canadian version): “Return to the start position. Now, imagine the movement, concentrate on the clarity of the image.” Secondly, for the kinaesthetic imagery ability evaluation patients have to concentrate on the intensity of the sensations during the imagination of all movements (original instructions from the Canadian version): “Now imagine the movement, concentrate on the intensity of the sensations.” After this instruction, patients imagined the movement and rated the vividness or sensation on the 5-point scale, respectively. All items were evaluated while sitting in a standardised sequence for visual and kinaesthetic scales (Table 2). The questionnaire requires a standardised procedure: 1) the examiner shows the movement once, 2) the patient performs the just seen movement once from a standardised starting position, 3) the patient imagines the movement once from the internal perspective, and 4) the patients scores the vividness of the ‘inner picture’ on a 5 point Likert scale (1 = ‘no image’, 5 = ‘image as clear as actually seeing it’) as well as the feeling associated with the imagined movements (1 = ‘no sensation’, 5 = ‘as intense as making the movement’). Patients were presented the evaluating words of the rating scale only. KVIQ-G scores were calculated for the short (10 items, range 5 to 25 points for each subscale, total score range 10 to 50) and the long (20 items, range 10 to 50 points for each subscale, total score range 20 to 100) version for the visual and kinaesthetic subscales.

**Table 2:** Movements for the short and long KVIQ-G version for both subscales

**Movements**	**KVIQ-G 20 visual**	**KVIQ-G 20 kinaesthetic**
Bend / stretch the neck	1 V	1 K
Shrugging the shoulders	2 V	2 K
**Lift the arm completely**	3 Vnd	3 Knd
Bend the elbow	4 Vd	4 Kd
**Touch the fingertips with the thumb**	5 Vd	5 Kd
**Bend the body forwards**	6 V	6 K
Stretch out the knee	7 Vnd	7 Knd
**Move the leg to the side**	8 Vd	8 Kd
**Tap the foot**	9 Vnd	9 Knd
Turn the foot outwards	10 Vd	10 Kd

**Legend:** Movements in bold represent the short version of the KVIQ-G visual and kinaesthetic subscales. KVIQ-G 10 or 20 = Kinaesthetic and Visual Imagery Questionnaire German version – short or long version, v = visual, K = kinaesthetic, nd = non-dominant side, d = dominant side. Movements with ^*^ = for bilateral assessment of limb movements. Not considered in the scoring for each subscale.

### Translation process

Both questionnaires are not self-administered. An examiner is involved in the questionnaire administration and movement assistance. Therefore, both questionnaires were forward and backward translated based on the guidelines for objectively-assessed outcome measures including a eight-step procedure [13]. The procedure included (1) an independent forward translation, (2) a review on adequate vocabulary by a person of the target profession, (3) a review on layout, grammar and typography, (4) a backward translation, (5) a review of all translations (forward, backward) by the original authors, (6) a re-check on layout, grammar and typography, (7) a pre-test with one patient and three examiners. The current work fulfils step number (8): a patient study to determine quality factors from the translated assessments. Permissions to use the translated assessments in this study were obtained from the original authors. The translated German versions of the questionnaires were termed Imaprax-G and KVIQ-G. During the translation process no instruction content were changed, added, or omitted.

### Participants

Participants were recruited from a larger ongoing intervention study [14] and from inpatients of the rehabilitation centre if they were older than 18 years, understood the study and were able to communicate verbally, and gave written informed consent. Further specific inclusion criteria had to be fulfilled: patients were included after a first-ever cerebrovascular accident (CVA), or intracerebral hemorrhage (ICB), or subarchnoid hemorrhage (SAB), or after a brain tumour surgery, with a consolidated diagnosis of Multiple Sclerosis, or Parkinson’s disease. Time between disease onset and study inclusion was not constrained. Patients were excluded when they had additional neurological and psychiatric diseases, or limb amputations.

### Study procedure and test administration

Patients were screened for study eligibility and received oral and written study information. After giving written informed consent patients were scheduled for two measurement events (T0, T1) within an interval of seven days. If possible, the measurement events were scheduled on the same time of the day with the same examiner. Before performing both MI assessments, patients rated how they felt on this day on an 11-point visual analogue scale (VAS), where the scale ends had been represented using smilies. A smiley with a big smile indicated high well-being (zero points) and a sad and crying smiley indicated low well-being (ten points). On both occasions patients received the German version of the Imaprax questionnaire before the KVIQ-G for two reasons: firstly, to evaluate the patients’ understanding of the MI concept, and secondly, to not influence their free selection of the MI perspective for Imaprax. With the KVIQ-G, patients evaluated all movements using the visual MI scale before using the kinaesthetic MI scale. If a patient was unable to perform the required movement due to a paresis, the examiner assisted to complete the movement. If assistance was necessary the examiner stood behind the patient or at the affected side of the body. The examiner gripped the patient’s upper or lower extremity from below to support normal movement without dominating or occluding the patient’s view on her/his own extremity by the examiners hands. The performance of both assessments took about 45 minutes. The study was approved by the responsible ethics committee (Kanton Aargau, Switzerland) as part of a larger project.

### Examiner experience

In this project patients were supported by four examiners. Three of them were physiotherapists with more than ten years of working experience. Two of them were holding a Master’s degree and one a Bachelor degree. The fourth examiner was a final year Master student of sports education and movement sciences. All examiners were trained by the first author to become familiar with the test administration and patient handling. The training included one hour of direct instruction, twice assistance during patient testing and twice supervision during own test administration with patients. Regular meetings during the study implementation ensured consistency in test administration.

### Analyses

**Descriptive data** were calculated representing frequencies, means, and standard deviations for patient’s personal and questionnaire data. For this analysis, patients were classified into five groups according to their rehabilitation stage and diagnosis: 1) subacute group (N = 17) – including all patients between 20 to 365 days after event onset with a CVA, ICB, SAB, brain tumour but without a lesion on the left parietal lobe, 2) chronic group (N = 34) – including all patients more than 365 days after event onset with a CVA, ICB, SAB without a lesion in the left parietal lobe, 3) left parietal lobe group (LPI, N = 7) – including all patients with a CVA, ICB or SAB that involved the left parietal lobe, 4) a Multiple Sclerosis group (N = 7), and 5) a Parkinson’s disease group (N = 8). Additionally, data are provided for the whole study population.

Additionally to the calculation of the scores for the short, long, and total **KVIQ-G,** scores were calculated for the dominant and non-dominant side with item: 3, 4, 5, 7, 8, 9, 10 of each side (range 7 to 35 points), upper (2x item 3, 4, 5 = range 6 to 30 points), and lower limbs (2x item 7, 8, 9, 10 = range 8 to 40 points). The **Imaprax** visual scale (vividness) has been summed up for all six gestures (minimal score 6 to maximal score 42). All questionnaire calculations were performed for both measurement events (T0, T1).

To determine **test retest reliability** the Intraclass Correlation Coefficients (ICC_1,1_) with a 95% confidence interval (CI) were computed for each questionnaire subscale (Imaprax-G: visual subscale; KVIQ-G: visual and kinaesthetic subscale; short and long KVIQ-G version) total scores (added scores of visual and kinaesthetic subscales), patient groups, and for all patients. The one-way analysis of variance random effect model was applied. **Internal consistency** (Cronbach’s α) was calculated for all questionnaire subscales at T0. CIs were determined by using the two-way analysis of variance random effect in the reliability analysis. Experts suggest to report the standard error of measurement (**SEM**) and ICCs for a questionnaire or assessment [15,16]. SEM and minimal detectable change (**MDC**) are clinically important measures to inform the clinician whether the changes in scoring of the patient are a variation of the measurement or a true change [15]. SEM and the MDC were calculated using the following formulas: $SEM=s*\sqrt{1-r}$ (s represents the standard deviation of the questionnaire mean at T0) and $MDC=1.96*\sqrt{2}*SEM$[15,17].

**Concurrent validity** of the construct MI vividness was computed by using the visual subscales of KVIQ-G and Imaprax-G at T0. Due to the required use of the internal perspective when performing the KVIQ-G, only patients, who choose the internal perspective in at least four out of six Imaprax-G gestures, were included in this analysis (N = 19). The Spearman Rank Correlation Coefficient (r_s_) was calculated. As suggested by the original authors the bifactorial structure of the KVIQ-G questionnaire was investigated with a promax oblique rotation **factor analysis**[8]. All calculations were computed with the SPSS version 19 (IBM company, USA) except for the SEM and MDC values, which were calculated using Microsoft Excel.

## Results

### Translation process

Table 2 provides an overview on all KVIQ-G movements (long and short version). Two movement descriptions were modified for easy and exact patient instruction in German: lift arm completely (original: forward shoulder flexion) and move the leg to the side (original: hip abduction).

### Patient description and questionnaire scores

All descriptive parameters, VAS, KVIQ-G visual and kinaesthetic scores as well as Imaprax-G scores of the 73 participants at T0 and T1 are displayed in Table 3. An overview on patients’ brain lesions is provided in Table 4. At T0 and T1 patients with Parkinson’s disease provided the lowest rating among all patient groups, whereas the chronic group at T0 and the MS group at T1 rated their well-being at the highest of all groups. At T0 the subacute group scored their visual and kinaesthetic imagery performance higher than any other group in the KVIQ-G subscales and Imaprax-G questionnaire. Conversely, the MS group showed the lowest scores in all MI assessments at T0. At T1 the LPI group scored highest in the KVIQ-G visual and Imaprax questionnaires, and the subacute group in the kinaesthetic subscale of the KVIQ-G. Interestingly, the LPI group scored above average in all MI assessments at T0 and T1.

**Table 3:** Descriptives, KVIQ-G and Imaprax-G value overview for all patient groups

**Diagnosis**		**Subacute group N = 17**	**Chronic group N = 34**	**Left parietal lobe group N = 7**	**Multiple Sclerosis N = 7**	**Parkinson’s disease N = 8**	**Total N = 73**
Age		65.0 (15.7)	62.5 (10.2)	61.6 (12.9)	48.0 (11.0)	73.4 (10.5)	62.8 (13.1)
Gender (females)		8	9	3	5	3	28
Handedness before disease onset (right)		17	32	5	7	7	68
Affected body side (right)		6	17	7	N/A	N/A	30 (16 N/A)
Time since disease onset (years)		0.2 (0.2)	3.5 (3.4)	2.3 (1.8)	14.8 (11.2)	5.2 (3.4)	3.9 (5.7)
VAS-1 sensitivities		3.0 (2.2)*	1.9 (1.8)*	4.1 (2.5)*	2.0 (2.0)	4.5 (2.3)	2.7 (2.2)
Test KVIQ-G 10 (25)	Vis	21.8 (4.0)	20.6 (4.7)	23.6 (1.4)	19.1 (7.1)	21.0 (2.9)	21.1 (4.5)
	Kin	20.8 (4.0)	16.9 (6.4)	19.7 (6.7)	14.3 (8.0)	21.0 (2.8)	18.3 (6.1)
Test KVIQ-G 20 (50)	Vis	43.6 (7.8)	40.7 (9.5)	46.6 (4.2)	37.7 (14.0)	41.3 (6.6)	41.7 (9.1)
	Kin	40.8 (8.4)	33.8 (12.6)	39.1 (13.7)	29.6 (16.3)	41.4 (6.9)	36.4 (12.1)
Test KVIQ-G total (100 P) (vis. + kin. subscale)		84.4 (13.7)	74.4 (20.3)	85.7 (14.0)	67.3 (15.1)	82.6 (13.3)	78.1 (17.9)
Test Imaprax-G (42)	Vis	33.6 (3.6)	32.7 (3.7)	32.3 (5.1)	31.1 (4.1)	32.3 (3.1)	32.7 (3.8)
VAS-2 sensitivities		3.7 (2.2)	2.6 (2.1)*	2.3 (1.7)*	2.3 (2.1)	4.9 (2.3)	3.1 (2.2)
Retest KVIQ-G 10 (25)	Vis	20.6 (4.4)	19.6 (5.2)	22.7 (2.8)	20.7 (3.4)	19.8 (4.8)	20.2 (4.6)
	Kin	20.1 (4.1)	16.0 (5.8)	19.8 (7.0)	15.4 (7.6)	20.3 (3.0)	17.8 (5.8)
Retest KVIQ-G 20 (50)	Vis	41.3 (8.0)	38.9 (10.1)	45.1 (5.4)	41.0 (6.9)	39.1 (10.2)	40.2 (9.0)
	Kin	39.9 (8.1)	31.7 (11.8)	39.3 (13.8)	30.4 (14.9)	41.3 (6.7)	35.3 (11.7)
Retest KVIQ-G total (100) (vis. + kin. subscale)		81.2 (15.1)	70.6 (20.0)	84.4 (13.7)	71.0 (17.0)	80.4 (16.0)	75.5 (18.2)
Retest Imaprax-G (42)	Vis	32.9 (4.5)	31.2 (5.5)	34.7 (4.1)	30.7 (2.8)	33.7 (1.6)*	32.1 (4.8)

**Legend:** Numbers represent means or frequencies. Numbers in brackets indicate standard deviation of the mean. N = sample size, KVIQ-G 10 or 20 = Kinaesthetic and Visual Imagery Questionnaire German version – short (10 items each body side) or long (20 items each body side) version, Imaprax-G = Imaprax German version, vis = visual subscale, kin = kinaesthetic subscale, N/A = not applicable, * = Data from 1 patient is missing.

**Table 4:** Location of patients’ brain lesions

**Right sided lesions N = 31**		**Left sided lesions N = 27**	
Lesion location	Amount of patients	Lesion location	Amount of patients
Basal ganglia	4	Basal ganglia	4
Cerebellum	1	Brain stem	3
Frontal cortex	2	Dorso-parietal cortex	1
MCA territory	15	Frontal cortex	2
Medulla oblongata	1	MCA territory	11
Occipital cortex	2	Medulla oblongata	1
Parietooccipital cortex	2	Occipital cortex	3
Tectum mesencephali	1	Parietooccipital cortex	1
Temporal cortex	2	Temporal cortex	1
Thalamus	1		

**Legend:** MCA = middle cerebral artery.

In the Imaprax-G (visual subscale) and KVIQ-G (visual + kinaesthetic subscales, short, long, and total versions) scoring patients did not show any floor or ceiling effects. Their scoring showed a trend to score in the upper third of the scoring range. All patients showed capacity for an increase or decrease in assessment scoring.

### Patients’ selected MI perspective

It is believed that the internal perspective is more beneficial during MI training. During the third stage of the Imaprax-G questionnaire procedure patients had to select their preferred MI perspective used for imaging the current gesture. Table 1 provides an overview on the selected internal MI perspectives for all groups for all six gestures at T0 and T1. The majority of patients selected the external perspective at both measurement events. The internal perspective was used at T0 and T1, especially in patients in the LPI and Parkinson’s disease groups. Table 5 provides a more detailed overview on right or left handed patients with their hemiparesis on the dominant side (dominant side affected), and left-handed patients with hemiparesis right (non-dominant side affected). Values for Imaprax-G visual and KVIQ-G 20 visual and kinaesthetic subscales for upper and lower limbs as well as for dominant and non-dominant side at T0 and T1 were added.

**Table 5:** Frequency analysis of chosen internal or external perspective in the Imaprax-G questionnaire at both measurement events

**Imaprax gestures**			**Right handed patients with hemiparesis right (dominant side affected) N = 26**	**Right handed patients with hemiparesis left (non-dominant side affected) N = 28**	**Left handed patients with hemiparesis right (non-dominant side affected) N = 4**	**Multiple Sclerosis group N = 7**	**Parkinson’s disease group N = 8**
1	To beckon somebody	Internal	5/5	8/9	2/2	2/1	4/4
		External	21/20	20/19	2/2	5/6	4/4
2	To cut something	Internal	11/11	13/15	3/4	3/2	6/4
		External	15/14	15/13	1/0	4/5	2/4
3	To write something	Internal	8/8	14/15	3/4	2/3	4/5
		External	18 17	14/13	1/0	5/4	4/3
4	To brush one’s teeth	Internal	4/5	7/8	1/2	3/2	3/3
		External	22 20	21/20	3/2	4/5	5/6
5	To cock a snook	Internal	4/6	9/8	2*/3	2/2	3/3
		External	22/19	19/20	1/1	5/5	5/5
6	To applaud somebody	Internal	9/8	13/14	4/4	4/2	3/3*
		External	17/17	15/14	0/0	3/5	5/4*
Imaprax-G visual			3.01 (3.8)	3.0 (3.7)	30.8 (5.0)	31.1 (4.1)	33.7 (1.6)
KVIQ-G 20 visual		Upper limb (30)†	25.0 (5.3)	25.0 (5.6)	25.0 (5.0)	22.0 (8.1)	25.3 (4.0)
		Lower limb (40)	34.2 (7.1)	34.1 (7.5)	37.0 (3.8)	32.9 (11.6)	32.6 (6.5)
		Dominant side (35)	28.9 (7.0)	31.0 (6.2)	32.5 (2.4)	27.7 (9.6)	29.9 (5.4)
		Non-dominant side (35)	30.3 (5.6)	28.2 (7.7)	29.5 (6.9)	27.1 (10.0)	28.0 (5.8)
KVIQ-G 20 kineasthetic		Upper limb (30)	23.4 (7.4)	21.1 (7.3)	15.5 (7.4)	18.0 (9.9)	25.0 (4.1)
		Lower limb (40)	31.7 (10.0)	29.4 (9.4)	20.0 (9.8)	24.9 (13.0)	33.4 (5.5)
		Dominant side (35)	26.5 (8.8)	26.4 (8.5)	17.8 (9.0)	21.4 (11.2)	29.0 (4.7)
		Non-dominant side (35)	28.7 (9.2)	24.1 (9.1)	17.8 (8.2)	21.4 (11.8)	29.4 (4.9)

**Legend:** The study population did not include left handed patients with a hemiparesis on the left body side (dominant side affected). Numbers represent means and standard deviations in brackets. N = sample size, † indicated maximal value, * information of one patient missing.

### Test-retest reliability

**Figure 1:**
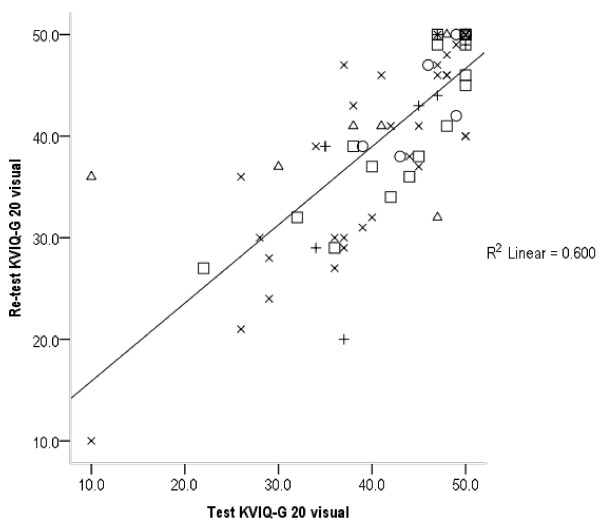
**KVIQ-G 20 visual subscale values at T0 and T1 (73 patients).** KVIQ-G 20 = Long version of the kinaesthetic and visual imagery questionnaire German version, R^2^ = Explaining variance of 60%, O = LPI group, □ = Subacute group, X = Chronic group, Δ = Multiple Sclerosis group, + = Parkinson’s disease group.

**Figure 2:**
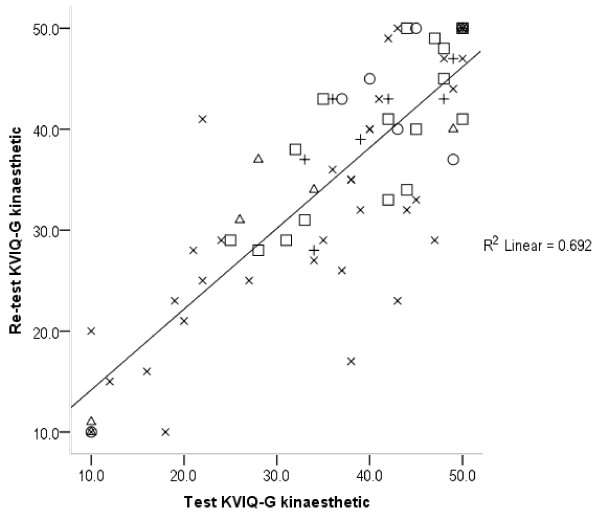
**KVIQ-G 20 kinaesthetic subscale values at T0 and T1 (73 patients).** KVIQ-G 20 = Long version of the kinaesthetic and visual imagery questionnaire German version. R^2^ = Explaining variance of 69%, O = LPI group, □ = Subacute group, X = Chronic group, Δ = Multiple Sclerosis group, + = Parkinson’s disease group.

**Figure 3:**
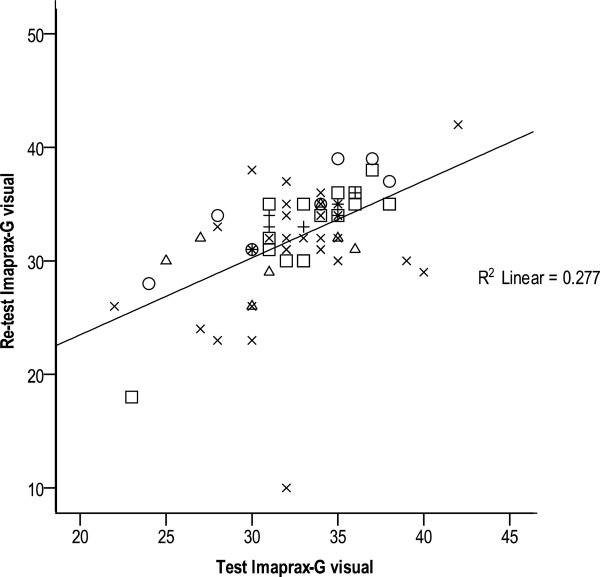
**Imaprax-G visual scale values at T0 and T1 (73 patients).** R^2^ = Explaining variance of 28%, O = LPI group, □ = Subacute group, X = Chronic group, Δ = Multiple Sclerosis group, +=Parkinson’s disease group.

An overview on all KVIQ-G and Imaprax-G subscale ICCs, their responsible CI for all patient groups is provided in Table 6. KVIQ-G kinaesthetic subscales (long and short version) revealed the highest ICCs in patients with MS (ICC: 0.95 and 0.92) compared to the chronic group, who show the lowest ICCs (0.75 and 0.80). On the contrary, the MS group showed the lowest ICCs in the visual subscale (0.43 and 0.51). The LPI group showed the highest ICC values in the visual subscales for long, short, and total KVIQ-G (vis + kin), which varied between 0.86 and 0.92. ICC values for the combined KVIQ-G are stable on a high level ranging between 0.79 and 0.88. Imaprax-G ICCs show a high variability ranging from 0.33 for chronic to 0.84 for subacute patient group. Figures 1, 2 and 3 visualise the association between test and retest values for the visual and kinaesthetic KVIQ-G subscales (long version), and Imaprax-G.

**Table 6:** Test retest reliability for the KVIQ-G subscales for the short and long version and the Imaprax-G visual subscale

		Subacute group N = 17*	**Chronic group N = 34**†	**Left parietal lobe involved N = 7**†	**Multiple Sclerosis N = 7**	**Parkinson’s disease N = 8**	**Total N = 73**
ICC KVIQ-G 10 (25)*	**vis**	**0.86**	**0.82**	**0.62**	**0.51**	**0.69**	**0.77**
95 % CI		0.66 – 0.95	0.67 – 0.90	−0.10 – 0.92	−0.67 – 0.94	0.10 – 0.89	0.66 – 0.85
ICC KVIQ-G 10 (25)*	**kin**	**0.79**	**0.80**	**0.88**	**0.92**	**0.84**	**0.85**
95 % CI		0.51 – 0.92	0.64 – 0.89	−0.52 – 0.98	0.66 – 0.99	0.44 – 0.97	0.77 – 0.90
ICC KVIQ-G 20 (50)*	**vis**	**0.83**	**0.84**	**0.77**	**0.43**	**0.68**	**0.77**
95 % CI		0.60 – 0.94	0.71 – 0.92	0.20 – 0.96	−0.35 – 0.87	0.08 – 0.93	0.65 – 0.85
ICC KVIQ-G 20 (50)*	**kin**	**0.80**	**0.75**	**0.91**	**0.95**	**0.82**	**0.83**
95 % CI		0.54 – 0.92	0.56 – 0.87	0.61 – 0.98	0.75 – 0.99	0.39 – 0.96	0.74 – 0.89
**ICC KVIQ-G total (100)**	**vis + kin**	**0.85**	**0.87**	**0.84**	**0.79**	**0.86**	**0.87**
95 % CI		0.64 – 0.94	0.77 – 0.94	0.38 – 0.97	0.26 – 0.96	0.48 – 0.97	0.80 – 0.92
**ICC Imaprax**	**vis**	**0.84**	**0.34**	**0.77**	**0.37**	**0.74**	**0.51**
95 % CI		0.62 – 0.94	0.005 – 0.60	0.19 – 0.95	−0.40 – 0.85	0.14 – 0.95	0.32 – 0.66

**Legend:** N = sample size, vis = visual subscale, kin = kinaesthetic subscale, ICC = Intraclass correlation coefficient, CI = confidence interval, ^*^ maximum score.

### Internal consistency and validity

Values for internal consistency measured with Cronbach’s α, SEM, and MDC are displayed in Table 7. Computed Cronbach’s α values revealed the highest estimate for the kinaesthetic KVIQ-G subscale (long version) and the lowest estimate for Imaprax-G with 0.70. SEM range between 0.3 for Imaprax-G and 4.0 for KVIQ-G total (vis + kin). Likewise, both questionnaires revealed the lowest and highest MDC values with 0.9 and 11.1 respectively.

**Table 7:** Internal consistency, SEM and MDC for all questionnaire subscales at T0

**N = 73**	**KVIQ-G 10 visual**	**KVIQ-G 20 visual**	**KVIQ-G 10 kinaesthetic**	**KVIQ-G 20 kinaesthetic**	**KVIQ-G total visual + kinaesthetic**	**Imaprax-G visual**
**Cronbach’s α**	0.88	0.94	0.92	0.96	0.95	0.70
**95% CI (two sided)**	0.83 – 0.92	0.92 – 0.96	0.88 – 0.94	0.94 – 0.97	0.93 - 96	0.57 – 0.79
**SEM**	1.56	2.2	1.73	2.4	4.0	0.3
**MDC**	4.3	6.2	4.8	6.7	11.1	0.9

**Legend:** KVIQ-G 10 or 20 = Kinaesthetic and Visual Imagery Questionnaire German version – short (10 items each body side) or long (20 items each body side) version, Imaprax-G = Imaprax German version, CI = confidence interval, SEM = Standard error of measurement ( $SEM=s*\sqrt{1-r}$), MDC = Minimal detectable change ( $MDC=1.96*\sqrt{2}*SEM$.

Spearman rank correlation coefficient revealed a low non-significant correlation between Imaprax-G visual subscale and the KVIQ-G visual subscale for the short (r_s_ = 0.32, p = 0.18) and long version (r_s_ = 0.36, p = 0.13). The factor analysis confirmed the bifactorial structure of the KVIQ-G and identified two factors to distinguish clearly between visual and kinaesthetic subscale with a correlation of r = 0.36. A third factor has been omitted due to a low correlation between factor 1 and 3, and factor 2 and 3 of r = 0.08. Factor 1 explains 49.2% and factor 2 explains 20.5% of the total variance. The Kaiser-Meyer-Olkin measure of sampling adequacy was 0.89 and the Bartlett test of sphericity was determined with p > 0.001. The pattern and structure matrix of the factor analysis are provided in Table 8.

**Table 8:** Principal factors solution with promax rotation of the KVIQ-G 20

**Subscale**	**Movements**	**KVIQ-G 20 items**	**Pattern matrix**		**Structure matrix**		**Communality**
			**Factor 1**	**Factor 2**	**Factor 1**	**Factor 2**	
Kin	Shoulder shrugging	2 K	0.93		0.90	0.24	0.82
	Knee extension	7Knd	0.90		0.87	0.23	0.85
	Neck flexion / extension	1 K	0.87		0.86	0.31	0.77
	**Hip abduction**	8 Kd	0.86		0.86	0.32	0.75
	**Thumb-fingers opposition**	5 Kd	0.85		0.85	0.31	0.73
	**Foot tapping**	9 Knd	0.84		0.86	0.33	0.78
	**Lift arm completely**	3 Knd	0.82		0.84	0.34	0.75
	Elbow flexion / extension	4 Kd	0.81		0.84	0.37	0.73
	**Forward trunk flexion**	6 K	0.80		0.84	0.39	0.71
	Foot external rotation	10 Kd	0.79		0.81	0.39	0.85
Vis	Foot external rotation	10 Vd		0.91	0.28	0.87	0.86
	**Hip abduction**	8 Vd		0.87	0.23	0.83	0.71
	Elbow flexion / extension	4 Vd		0.86	0.34	0.86	0.78
	**Thumb-fingers opposition**	5 Vd		0.84	0.35	0.85	0.73
	**Forward trunk flexion**	6 V		0.80	0.27	0.79	0.63
	Neck flexion / extension	1 V		0.78	0.40	0.82	0.69
	Knee extension	7 Vnd		0.75	0.37	0.81	0.81
	**Foot tapping**	9 Vnd		0.73	0.33	0.78	0.73
	Shoulder shrugging	2 V		0.69	0.32	0.73	0.60
	**Lift arm completely**	3 Vnd		0.68	0.34	0.74	0.76

**Legend:** Words in bold in the movement column indicate movements that belong to the short KVIQ-G version (KVIQ-G 10). KVIQ-G 20 = Kinaesthetic and Visual Imagery Questionnaire German version - long version (20 items), Vis = visual subscale, Kin = kinaesthetic subscale, d = dominant side of the body, nd = non-dominant side of the body.

## Discussion

The results of our study revealed very good (0.8 < ICC < 0.9) test retest reliability values for all patient groups for the visual and kinaesthetic subscales of the KVIQ-G short and long version [18]. Exceptions showed MS and Parkinson’s disease groups for the visual KVIQ-G subscale in both versions. ICCs for the total KVIQ-G (vis. + kin.) showed a very good overall test retest reliability for all groups.

Patients in the chronic group and MS patients produced unacceptable (below 0.6) Imaprax-G ICCs that lead to an unacceptable overall ICC according to DeVellis [18]. Nevertheless, the subacute, PLI, and Parkinson’s disease groups produced very good and respectable test retest reliability values.

Based on the original literature by Malouin et al. (2007) no group differences were expected [8]. Regardless the higher average MI vividness scores in the KVIQ-G (KVIQ-G short: 1.4-2.7 points, KVIQ-G long: 3.6-6.6 points, KVIQ-G total: 8.0-11.9 points) compared to the work of Malouin et al. our analyses computed slightly lower ICC values showing a difference from 0.01 to 0.15 compared to Malouin’s reported ICCs for CVA patients [8]. The higher KVIQ-G values could be explained by the educational effect of the questionnaire order applied and its resulting longer MI vividness evaluation duration. Likewise, the KVIQ-G subgroup ICC values of our Parkinson’s disease group were lower (0.01-0.14) than in the values reported for KVIQ in the work of Randhawa et al. (2010) [10]. Nevertheless, values were higher in the total KVIQ-G compared to values in [10]. At T0 patients in the MS group showed the lowest KVIQ-G scores for the visual and kinaesthetic subscales in short or long version as well for the Imaprax-G. This remained the same for T1 except for the short and long visual KVIQ-G subscale. We hypothesise that the below average scoring could be related to the longest disease duration of 14.8 years on average, compared to the other patient groups. Overall, KVIQ-G scores were higher for the visual subscale than for the kinaesthetic subscale at T0 and T1, and higher for the affected side than for the non-affected side, which corresponds to the reports of Malouin et al. and Randhawa et al. [8,10,19]. Additionally, KVIQ-G 20 values for Parkinson’s disease were about 10 points below the ones reported by Randhawa and colleagues for visual and kinaesthetic subscale [10]. This could be due to the longer disease duration of more than 4.6 years in the current study. MS patients were the only patient group, whose KVIQ-G scores increased for all versions between 0.8 and 3.3 from T0 to T1. Scores of all other patient groups decreased from T0 to T1 for all KVIQ-G versions between 0.5 and 1.5. Several reasons could have contributed to this change. While aiming for constant test conditions, we are aware of day-to-day differences or medication dependencies of the questionnaire results. Furthermore, the construct of MI is very abstract. At T1 patients were more familiar with the topic and rated themselves more critically. We hypothesised that there will be a learning effect for all assessments and questionnaires that will be performed more than once with the same patient or healthy person. In the present study, we believed that patients did learn about the evaluation procedure and the construct of MI, which most of them did not know before. We do not believe that patients learned all movements or gestures they had to imagine, or remembered the different rating scales for KVIQ in detail, or the Imaprax video they had chosen during the first time.

Our SEM values for all patients were similar to the reported values from Malouin et al. for CVA patients in the visual and kinaesthetic subscales of the short KVIQ-G version [8]. Both KVIQ-G subscales revealed clearly lower SEM values for the long version.

Cronbach’s alpha values for internal consistency showed higher values (0.88 to 0.96) compared to Malouin and colleagues (2007) indicating a high item homogeneity for the construct assessment of MI vividness [8].

Our concurrent validity testing did not result in a high correlation value as expected. We assume that the correlation result is influenced by the number of patients available for this analysis rather than differences between KVIQ and Imaprax in the construct MI vividness. KVIQ-G required patients to assume an internal perspective, whereas Imaprax-G let patients choose the MI perspective. As a result, only 19 patients did select the internal MI perspective for both questionnaires and could therefore be included in the validity analysis.

To provide further inside in the structure of the KVIQ-G a factor analysis was performed. In the statistic literature different item participant ratios for exploratory factor analysis are suggested varying between 1 vs. 3 to 1 vs. 10 [20]. Our successfully performed analysis with a ratio of 1:3.7 confirmed the bifactorial structure of the KVIQ-G.

### The MI perspective

The Imaprax-G let patients freely select a MI perspective for each of the six gestures. Frequency analyses showed that the minority of the patients, whether in a disease group, or classified for dominant or non-dominant hand affect, selected the internal perspective. A possible explanation for the phenomenon could be found in Li (2000), who observed an impaired MI of limbs in the internal, but not in the external MI perspective [21]. Furthermore, Kim et al. (2009) investigated the exercise-related imagery perspective in middle-age adults and reported an internal vs. external perspective ratio of 1.8 [22]. Mulder et al. (2007) could show a slightly better MI vividness in adults over 64 years when using the external MI perspective [23]. The KVIQ-G requires patients to use the internal MI perspective but did not consider testing for a patient’s understanding of the MI perspective component. Therefore, we recommend determining the spontaneously selected MI perspective in patients or their ability to distinguish between both MI perspective options, before administering the KVIQ-G or any other MI vividness assessments. This could be done by using the Imaprax-G questionnaire or ask the patients what they have seen in their ‘inner picture’ after performing an MI gesture or movement example.

### The parietal lobe debate

Various studies discussed the involvement of the right and left parietal lobe in MI and attributed the parietal cortex to be responsible for MI movement generation and preservation [24-26]. After stroke aspects of this function can be affected resulting in impaired MI performance. Sack et al. (2005) found evidence that the right parietal lobe may overtake left parietal functions [27]. Considering the results of our LPI group no divergently KVIQ-G or Imaprax-G values could be detected. Therefore, we are suggesting to not excluding patients from MI interventions based on a lesion in the left parietal lobe only. Nevertheless, patients MI vividness evaluation should not be limited to vividness only as tested with assessments, e.g. KVIQ, or Imaprax. Further procedures, e.g. mental chronometry, mental rotation, to evaluate different aspects of patients MI vividness should be used to confirm assessment findings in general [28].

### Study limitations

The small sample size in the MS (N = 7) and Parkinson’s disease (N = 9) groups raised two issues in the data analysis and reported results: Firstly, the CI lower bounds dropped to negative values and thus, the concurrent validity between KVIQ-G visual and Imaprax-G visual could not be determined. Nevertheless, in the original publication by Malouin et al. a subgroup with five participants with a lower limb mobilisation was investigated [8]. In the current investigation, presented results included results for different subgroups and the total study population. It is believed that the chosen sample size is helpful to detect potential differences. An inference statistical analysis was avoided.

Secondly, a low number of patients (N = 19) could be included in the validity correlation analysis, due to their selection of the internal MI perspective when administering the Imaprax-G questionnaire. Further aspects should be considered when interpreting the data: Firstly, the current study did not include a control group to compare MI questionnaire values with age-matched healthy controls. Based on the findings of Malouin et al., we did not expect variations in the assessment scoring between patients and healthy controls [8]. Secondly, Imaprax-G and KVIQ-G were always administered in the mentioned order. We avoided a random procedure to prevent influencing patients in their MI perspective selection for Imaprax-G.

## Conclusions

The successful translation process yielded German versions of the Imaprax and KVIQ assessments. Both questionnaires showed respectable to very good reliability values for visual and kinaesthetic subscales as well as for the short and long KVIQ-G version. Concurrent validity could not be determined by correlating values of both questionnaires, but the bifactorial structure of the KVIQ-G could be confirmed by a factor analysis.

KVIQ-G visual scores were larger than those for the kinaesthetic scores. Patients with MS showed the lowest, whereas the subacute group showed the highest questionnaire scores, which could be due to longer and shorter disease duration. Patients with a hemiparesis scored lower in both KVIQ-G subscales of the affected side compared to the non-affected side. Furthermore, Imaprax-G is the first MI vividness questionnaire that takes the patients’ selected MI perspective into account. Therefore, for practical reasons we suggest administering both assessments: the Imaprax-G before the KVIQ-G to explain and test the patient’s ability to distinguish between external and internal MI perspective. The internal MI perspective is required by the KVIQ-G to assess patients’ MI vividness. Furthermore, administering both assessments could lead to an educational effect resulting in higher scoring. For practical reasons and although the KVIQ-G long version achieved higher reliability values, we recommend to administer the short version for patients with a central lesion due to its lower time requirements of 20 to 25 minutes.

## Abbreviations

α: Cronbach’s alpha; ADL: Activities of daily living; CI: Confidence Interval; ICC: Intraclass correlation coefficient; Imaprax-G: German version of Imaprax; kin: Kinaesthetic; KVIQ: Kinaesthetic and Visual Imagery Questionnaire; KVIQ-10: Kinaesthetic and Visual Imagery Questionnaire short version; KVIQ-20: Kinaesthetic and Visual Imagery Questionnaire long version; KVIQ-G: German version of the Kinaesthetic and Visual Imagery Questionnaire; KVIQ-G-10: German version of the Kinaesthetic and Visual Imagery Questionnaire short version; KVIQ-G-20: German version of the Kinaesthetic and Visual Imagery Questionnaire long version; LPI: Left parietal lobe involved; MDC: Minimal detectable change; MI: Motor imagery; MIQ: Movement Imagery Questionnaire; MIQ-R: Revised Movement Imagery Questionnaire; N: Sample size; PP: Physical Practice; r_s_: Spearman Rank Correlation Coefficient; SEM: Standard error of measurement; T0: First measurement event; T1: Second measurement event; VAS: Visual analogue scale; Vis: Visual.

## Competing interests

The authors declare that they have no conflict of interests.

## Authors’ contribution

CSch made substantial contributions to conception and design, acquisition of data, analysis and interpretation, and wrote the manuscript. AL made substantial contributions to conception and design, acquisition of data, analysis and interpretation, was involved drafting the manuscript and revising it critically for important intellectual content. BW made substantial contributions to conception and design, data interpretation and revising the manuscript critically for important intellectual content. ThE made substantial contributions to conception and design, and revised the manuscript critically for important intellectual content. All authors gave final approval of the version to be published.

## Pre-publication history

The pre-publication history for this paper can be accessed here:

http://www.biomedcentral.com/1471-2288/12/127/prepub
